# Validation of the Serbian Version of Multiple Sclerosis Spasticity Scale 88 (MSSS-88)

**DOI:** 10.1371/journal.pone.0147042

**Published:** 2016-01-15

**Authors:** Sindi Z. Rodic, Tatjana I. Knezevic, Darija B. Kisic-Tepavcevic, Jelena R. Dackovic, Irena Dujmovic, Tatjana D. Pekmezovic, Jelena S. Drulovic, Ljubica M. Konstantinovic

**Affiliations:** 1 Clinic for rehabilitation Dr M. Zotovic, Belgrade, Serbia; 2 University Chlidren's Hospital, Belgrade, Serbia; 3 Faculty of Medicine, University of Belgrade, Serbia; 4 Institute of epidemiology, Belgrade, Serbia; 5 Clinic for Neurology, Clinical Centre of Serbia, Belgrade, Serbia; San Raffaele Scientific Institute, ITALY

## Abstract

**Objective:**

Multiple Sclerosis Spasticity Scale (MSSS)-88 has been developed for self-assessment of spasticity symptoms in patients with multiple sclerosis (MS). The objective of this study was to validate MSSS-88 and evaluate the psychometric properties in patients with MS in Serbia.

**Methods:**

The study comprised 65 MS patients with spasticity. MSSS-88 consists of 88 items grouped in eight sections. Internal consistency of the MSSS-88_SR_ subscales was determined using Cronbach’s alpha coefficient. Test/retest reliability with an intra-class correlation coefficient (ICC) for each MSSS-88_SR_ subscale was performed. Clinical validity of MSSS-88_SR_ was determined by correlations with the Numeric Rating Scale (NRS) and the Modified Ashworth Scale (MAS).

**Results:**

The range of Cronbach’s alpha for all scales and ICC was 0.91–0.96 and 0.84–0.91, respectively. All ICCs were statistically significant (p<0.05). All evaluated subscales of MSSS-88 were significantly correlated with the NRS scale. The highest correlation coefficients were registered between the WL subscale and the EDSS and MAS, while the strongest relationship was observed between the MSS subscale and the NRS.

**Conclusion:**

The Serbian translated version of this instrument may be useful as a clinical measure for spasticity and functionality in patients with MS.

## Introduction

Multiple sclerosis (MS) is one of the most common chronic, progressive neurologic diseases affecting young adults. MS leads to considerable disability in daily life, and is unpredictable with impairments and activity limitations that can appear suddenly and progress over time [1]. The common functional limitations and symptoms of MS are associated with disorders of strength, sensation, coordination, and balance, as well as cognition, mood, and vision disturbances [2]. Spasticity is one of the most common symptoms in MS patients, and increases in severity as the disease progresses [3]. It has been demostrated that spasticity occurs in up to 85% of MS patients [4,5]. Spasticity is defined as a motor disorder characterized by a velocity-dependent increase in tonic stretch reflexes and exaggerated tendon reflexes resulting from increased excitability of the stretch reflex [6]. Spasticity is usually accompanied by muscular weakness and is sometimes associated with a pain [7].

Spasticity can be measured through neurophysiologic, biomechanical, and clinical evaluations. The Modified Ashworth five-level scale (MAS), as part of the clinical assessment of spasticity, is a quick and easy way to measure muscle hypertonia. The MAS measures resistance during passive soft tissue stretching with the speed of gravity [8]. The validity, reliability, and sensitivity of this scale have been challenged and is not considered an ideal scale for assessing the severity of MS spasticity [9]. In patients with MS, apart from a complete psychophysical status and objective neurologic status, a subjective perception of symptoms and signs must be also considered. In the last few years, a number of subjective MS-specific measures of spasticity and the impact of spasticity on daily life have been developed. Recently, several of these MS-specific measures have been evaluated, including Multiple Sclerosis Spasticity Scale (MSSS)-88 [10], the Patient-reported Impact of Spasticity Measure [11], Multiple Sclerosis Impact Scale-29 [12], and the Multiple Sclerosis Walking Scale [13].

The MSSS-88 has recently been developed for the subjective assessment of spasticity symptoms and functional impact on eight clinically-separate domains [10]. The MSSS-88 comprises 88 items grouped in eight subscales. Thus, MSSS-88 has the potential to quantify the impact of spasticity based on three spasticity-specific symptoms (muscle stiffness, pain and discomfort, and muscle spasms), three areas of physical functioning (activity of daily life, walking, and body movements), emotional health, and social functioning. Therefore, as a patient-oriented instrument, MSSS-88 comprising these subscales has the potential for measuring the broader impact of spasticity in MS patients [6]. Furthemore, the advantage of MSSS-88 is that each subscale represents a stand-alone measurement instrument and clinicians may use MSSS-88 for different measurement purposes. Such a feature is important because spasticity management should be patient-focused. It is very important to clarify that other scales related to spasticity with few items have limited accuracy and are less able to detect small, but clinically-meaningful changes [10]. It has already been shown that MSSS-88 is sensitive to changes which could be of utmost importance in daily practice and clinical trials for assessment of treatment effects.

The main objective of this study was to evaluate the psychometric properties of MSSS-88 in Serbian patients with MS.

## Materials and Methods

### Patients

Included in the study were 65 patients with a diagnosis of MS (Mc Donald criteria) [14], who were referred to the Clinic for Rehabilitation (Dr. M. Zotovic). The inclusion criteria were as follows: ≥ 18 years of age; and presence of MS-related spasticity for at least 12 months. The exclusion criteria were as follows: spasticity related to other causes than MS; relapse of MS in the last 3 months; and cognitive impairment (Mini-mental score <24). All participants signed an informed consent approved by the Ethics Committee of the Faculty of Medicine of University of Belgrade.

### Development of the Serbian version of MSSS-88

The MSSS-88 was translated from English-to-Serbian [15, 16]. All items of the scale were first translated, then discussed by two independent translators. This version was translated back to English by a bilingual translator not involved in the forward translation. The discrepancies between the forward and backward translations were discussed among the three translators and three MS experts, and agreement was reached through consensus.

### Data collection

Demographics and clinical characteristics were collected through face-to-face interviews between the physician and patients. Structured documentation forms and questionnaires were used for data collection. The following demographic and clinical data were collected: age; gender; employment status; marital status; educational status; disease history; duration of the disease; and degree of spasticity within the last 24 hours according to the Numeric Rating Scale (NRS). All patients were assessed with the EDSS and the MAS.

### Validated instrument

The MSSS-88_SR_ was completed by the patient two times (test, on the first day of hospitalization [at the time of admission] and retest, 7 days later), with instructions provided by the same examiner. The scale consisted of 88 items grouped in eight sections, as follows: muscle stiffness (MSS), 12 items; pain and discomfort (PD), nine items; muscle spasms (MS), 14 items; activities of daily life (ADL), 11 items; walking (WL), 10 items; body movement (BM), 11 items; emotional health (EM), 13 items; and social functioning (SF), eight items. Each item was ranked on a 4-point Likert scale, as follows: 1 (not bothered at all); 2 (mildly bothered); 3 (moderately bothered); and 4 (extremely bothered). Each subscale of MSSS-88_SR_ is scored as a stand-alone measurement instrument. Developers of the scale offer three methods for computing the MSSS-88 subscale score [10]. Using the first method, item scores can be summed without weighting or standardization. Missing responses to scale can be replaced with the mean score of items completed (person–specific item mean score) provided that ≥ 50% of the items in a scale have been completed [17]. The summed scores generated above, using the second method of computing MSSS-88 subscale scores, can be transformed using conversion tables. Using the third method of computing MSSS-88 scores, clinicians can analyze their own data by Rasch analysis. In our study, we used the first method due to the number of patients. The total MSSS-88_SR_ is reported for descriptive purposes only because the value remains unknown.

### Validity

Convergent validity of MSSS-88_SR_ was determined against NRS for spasticity and against MAS [8,17]. NRS is a simple 11-point scale ranging from 0–10 (0 = no spasticity and 10 = the most severe spasticity). As a self-rated assessment, patients are asked to indicate the mean level of spasticity over the previous 24 h [18]. Ashworth scores ranging from 0–4, with a 6-point scale (0, 1, 1+, 2, 3, 4; 1+ assigned 1.5), in which knee extensors were evaluated bilaterally; the higher value of the two scores was used for interpretation. Scores for NRS and MAS were only administered during the first completion of the test.

### Statistical analyses

Cronbach’s alpha coefficient was used to analyse the internal consistency of the subscale. The reproducibility of scores was evaluated by calculation of the test/retest correlation (intra-class correlation coefficient [ICC]).

Statistical comparisons were performed between the means (medians) of scores and subscales for test and retest according to gender, education, Ashworth scale, and NRS (separately), Independent t-tests were used for two independent groups (Wilcoxon Mann–Whitney test for two independent groups). Comparisons of means (medians) between of test and retest scores and test and retest subscales were done using paired t-tests (Wilcoxon test).

The calculation of Spearman’s correlation coefficients (ρ) between test subscales and retest subscales were investigated separately. SPSS 17.0 (Chicago, IL, USA) was used for statistical analyses.

## Results

The results of descriptive statistics of all measured parameters are shown in Table 1.

**Table 1:** Patients characteristics.

Variable	MS patients
Number	65
Sex, *n* (%)	
Female	47 (72.3%)
Male	18 (27.3%)
Age (years) (mean ± SD)	46.7±9.2
Education, *n* (%)	
Secondary school	46 (70.8%)
University	19 (29.2%)
Marital status, *n* (%)	
Married	41 (63.1%)
Divorced	11(16.9%)
Single	13 (20.0%)
Employment, *n* (%)	
Retired	36 (55.4%)
Employed	22 (33.8%)
Unemployed	7 (10.8%)
Duration of MS (years) (mean ± SD)	10.4-±7.1
Disease course, *n* (%)	
Relapsing-Remitting	10 (15.38%)
Secondary Progressive	21 (32.31%)
Primary Progressive	34 (52.31%)
EDSS score (mean ± SD)	5.1±1.3
MAS (mean ± SD)	2.0 ±0.9
NRS (mean ± SD)	6.4 ±2.3

SD = standard deviation.

EDSS = Expanded Disability Status Scale score

MAS = Modified Ashworth Score

NRS = Spasticity Numeric Rating Scale.

The study participants included 47 women (72.3%) and 18 men (27.3%). The mean (±standard deviation) age of the patients was 46.67±9.18 years. Most of the patients were married (63.1%) and had a secondary school education (70.8%). The mean value of the degree of spasticity, as measured by the NRS, was 6.37±2.30. The mean EDSS score was 5.1±1.3 The mean estimated number of spasms per patient was 2.04 ±0.88.

The results of comparisons of means between subscale scores on the test and retest are shown in Table 2. There were no statistically significant differences between values registered in two time points.

**Table 2:** Differences in MSSS-88 subscale scores between test and retest.

Subscales		Mean±SD	p-value
MSS	Test	32.4±10.9	0.544
	Retest	31.8±12.1	
PD	Test	22.3±8.7	0.112
	Retest	21.4±9.5	
MS	Test	33.0±13.6	0.651
	Retest	33.1±15.1	
ADL	Test	24.6±10.8	0.509
	Retest	23.9±10.8	
WL	Test	29.4±8.2	0.090
	Retest	28.4±9.7	
BM	Test	28.3±11.3	0.434
	Retest	27.7±11.8	
EH	Test	27.4±12.0	0.060
	Retest	26.4±12.7	
SF	Test	16.9±7.2	0.277
	Retest	16.7±7.7	
Total	Test	214.1±72.7	0.314
	Retest	209.0±80.4	

MSS = Muscle Stiffness

PD = Pain/discomfort

MS = Muscle Spasms

ADL = Activity of Daily Life

WL = Walking

BM = Body Movements

EH = Emotional Health

SF = Social Functioning.

The Cronbach’s alpha and ICC for MSSS-88 subscale values between the test and retest are shown in Table 3. The range of Cronbach’s alpha for all scales was 0.913–0.956. The range for ICC for all subscales was 0.84–0.91. All intra-class correlation coefficients were statistically significant (p<0.05).

**Table 3:** Descriptive statistics with Cronbach’s alpha and intraclass coefficients for MSSS-88 subscales.

Subscale	Cronbach’s α	ICC	95% CI of ICC	
MSS	0.94	0.89	0.82	0.93
PD	0.93	0.87	0.80	0.92
MS	0.93	0.86	0.78	0.91
ADL	0.94	0.89	0.83	0.93
WL	0.91	0.84	0.75	0.90
BM	0.94	0.89	0.82	0.93
EH	0.96	0.91	0.86	0.95
SF	0.92	0.86	0.78	0.91

ICC = Intraclass coefficients

MSS = Muscle Stiffness

PD = Pain/discomfort

MS = Muscle Spasms

ADL = Activity of Daily Life

WL = Walking

BM = Body Movements

EH = Emotional Health

SF = Social Functioning

α—alpha

ICC–intra class correlation coeficient

95% CI—95% confidential interval.

The Spearman’s correlation coefficients for MSSS-88 subscales scores and the EDSS, MAS, and NRS scores are reported in Table 4.

**Table 4:** The correlation analysis of MSSS-88 subscales with the EDSS, MAS and NRS scales scores.

Subscales	EDSS		Ashworth scale		NRS scale	
	ρ	p-value	ρ	p-value	ρ	p-value
MSS	0.338	0.006	0.377	0.003	0.624	0.000
PD	0.205	0.102	0.164	0.191	0.395	0.001
MS	0.313	0.011	0.318	0.013	0.517	0.000
ADL	0.458	0.000	0.452	0.000	0.507	0.000
WL	0.551	0.000	0.535	0.000	0.619	0.000
BM	0.404	0.001	0.411	0.001	0.563	0.000
EH	0.170	0.177	0.151	0.229	0.350	0.004
SF	0.272	0.028	0.299	0.016	0.545	0.000

MSS = Muscle Stiffness

PD = Pain/discomfort

MS = Muscle Spasms

ADL = Activity of Daily Life

WL = Walking

BM = Body Movements

EH = Emotional Health

SF = Social Functioning

ρ = Spearman’s correlation coefficient.

The highest correlation coefficients were registered between the WL subscale and the EDSS and Ashworth scales, while the strongest relationship was observed between the MSS subscale and the NRS.

## Discussion

The purpose of this study was to translate the MSSS-88 instrument into the Serbian language and determine the validity and reliability as a tool for spasticity assesment. Spasticity is one of the most common disabling symptoms in patients with MS and has important implications in personal functionality and quality of life [19]. Assessing the severity of spasticity in daily practice requires valid and quantitative measures. Several biomechanical, electrophysiological, and clinical methods are recognized as the standard for evaluating the level of spasticity that correlate with functional measures. Indeed, an appropriate method for monitoring the degree of spasticity in the daily rehabilitation program for MS patients could be developed with an accurate assessment of spasticity.

The usefulness of MAS has been demonstrated as an objective spasticity evaluation [8]. In our study, the severity of spasticity was assessed by physicians and patients. Therefore, we used MSSS-88 as one of the reliable tests for evaluating the impact of spasticity on MS patients [10]. Assuming that aforementioned two scales comprise the objective and subjective measurement of spasticity, one could assume that the concurrent usage might lead to more comprehensive assessment of this clinical manifestation.

It has been demonstrated that different clinical indicators of spasticity have various degrees of sensitivity for estimation of the impact of spasticity in MS. The authors of MSSS-88 developed a reliable and valid patient-derived instrument for evaluating spasticity through three main domains (symptoms, physical functioning, and psychosocial functioning). Initially, MSSS-88 consisted of 144 items with 5-point response options for each item. The final and definite version of MSSS-88 had 88 items grouped in 8 subscales and each item was ranked on a 4-point Likert scale. Using an explicit mathematical model, the authors showed that MSSS-88 comprises a reasonable number of items and each subscale can be used as a stand-alone measurment instrument. Additionally, the authors suggest that clinicians and researchers use a short form version of MSSS-88 with choice questions adequate for each subscale. Using Rash analysis as a complicated and infrequently used model for psychometric characteristics is not recomended by authors of MSSS-88.

In this study we used the first recommended method, thus item scores were summed. For the defined subscales of MSSS-88, there were no statistically significant differences between test and retest values. These observations suggest that every item was sufficiently understandable for our patients. High internal consistency for every item of MSSS-88 (>0.90) was demonstrated in our analysis. Such findings are in agreement with previous reports and exceed the suggested minimum of 0.70 [20, 21].

All evaluated subscales of MSSS-88 significantly correlated with the NRS. These findings are similar to those in a previously published study [20].

The limitation of our study involves the small number of enrolled participants (N = 65). In contrast, it has to be emphasished that the response rate was 100% for the test and retest.

This study has shown that the MSSS-88 (Serbian version) may be used as a reliable and valid instrument for assessment of spasticity in patients with MS. As a patient-oriented test with excellent psychometric properties, the MSSS-88_SR_ could be used as an instrument to obtain additional information regarding spasticity and functionality. Therefore, this instrument might help health care professionals in the the follow-up of functional recovery and establishing an individualized training program for MS patients.
